# Murine mesenchymal progenitor cells from different tissues differentiated *via *mesenchymal microspheres into the mesodermal direction

**DOI:** 10.1186/1471-2121-10-92

**Published:** 2009-12-19

**Authors:** Florian Böhrnsen, Ulrich Lindner, Markus Meier, Abdelalim Gadallah, Peter Schlenke, Hendrik Lehnert, Jürgen Rohwedel, Jan Kramer

**Affiliations:** 1 Institute of Virology and Cell Biology, University of Lübeck, Ratzeburger Allee 160, 23538 Lübeck, Germany; 2 Institute for Transfusion Medicine and Transplantation Immunology, University of Münster, Domagkstraße 11, 48149 Münster, Germany; 3 Medical Department I, University of Lübeck, Ratzeburger Allee 160, 23538 Lübeck, Germany

## Abstract

**Background:**

Because specific marker molecules for phenotypical identification of mesenchymal stem and progenitor cells are missing, the assessment of the *in vitro*-differentiation capacity is a prerequisite to characterize these cells. However, classical differentiation protocols are often cell-consuming and time intensive. Therefore, the establishment of novel strategies for differentiation is one topic of current efforts in stem cell biology. The goal of this study was to demonstrate the practicability of a new differentiation test using plastic adherent cell isolates from different tissues.

**Results:**

We introduced the mesenchymal microsphere method as a feasible time- and cell saving screening method to analyse multilineage differentiation properties of adult progenitor cells in a three-dimensional system. For this purpose we isolated, characterized and analyzed new sources of adult murine mesenchymal progenitor cells from perirenal adipose tissue and mediastinal stromal tissue in comparison to bone marrow progenitor cells. The proliferation capacity of the cells was demonstrated by determination of the daily doubling index. Although the flow cytometry analysis of undifferentiated cells revealed differences in the expression of CD marker molecules, all isolates have the capacity for multilineage differentiation following the mesenchymal microsphere protocol as well as the classical "micro mass body" protocol for chondrogenic and the monolayer cultivation protocol for osteogenic and adipogenic differentiation. Differentiation was characterized using histochemical and immunhistochemical staining as well as RT-PCR.

**Conclusions:**

We were able to show that the mesenchymal microsphere method is an efficient test system for chondro-, osteo- and adipogenic differentiation of adult progenitor cells. The advantage of this system in comparison to classical protocols is that approximately 7 times lower cell numbers are necessary. Since classical culture procedures are time intensive because high cell numbers have to be obtained, the new differentiation method may also save cells and time in future clinical applications using human mesenchymal stromal cells.

## Background

Many tissues exhibit the capacity for renewal after trauma, disease or aging because of dormant stem cell reservoirs. Different types of stem cells have been described within the adult bone marrow including haematopoietic [1] and colony forming units-fibroblast-like-cells [2], later termed bone marrow stromal cells [3] which have been found to differentiate along multiple mesenchymal lineages [4-7]. Other sources of adult stem cells have recently been characterized, suggesting that stem cells capable of multilineage differentiation might reside in any post-natal organ [8]. However, their multilineage potential has often been considered to represent heterogenic cell isolates [9-11].

Three criteria have been chosen to define mesenchymal stromal cells [7]. First, the cells are plastic adherent and second, they express the CD marker molecules CD105 (endoglin), CD73 (5'-nucleotidase) as well as CD90 (Thy1) and do not express the leukocyte marker molecule CD45 and the marker molecule CD34 for primitive hematopoietic progenitor cells. However, current studies demonstrated that mesenchymal stromal cells cannot be distinguished from fibroblasts by flow cytometry analysis using a panel of common marker molecules [12]. Therefore, the third criterium plays a pivotal role: mesenchymal stromal cells must show the multilineage capacity to differentiate into adipogenic, osteogenic and chondrogenic cells.

It is well known, that isolation and proliferation as well as *in vitro*-differentiation tests of adult stem and progenitor cells are time consuming and therefore not easily applicable in clinical protocols. Two different strategies have been suggested to overcome this problem. First, autologous reparative cells can be guided directly *in vivo *to a defect by exogenous factors. For example, an orthopaedic method using a collagen I/III-matrix to recruit mesenchymal stem cells from the subchondral bone to a cartilage lesion after microfracture has been developed [5]. However, using this strategy under clinical conditions it remains unclear whether defined populations of stem/progenitor cells migrate to the defect. For this purpose, *in vitro *cultivation procedures have to be optimized to establish time-sparing protocols before defined human stem/progenitor cells can be used for regeneration of different organs in clinical settings. One goal of this strategy is to reduce the amount of the cells used for assessing the differentiation capacity. Therefore, we introduced the mesenchymal microsphere (MMS) cultivation system and tested the differentiation of plastic adherent cells from different sources, namely murine perirenal adipose tissue (PAT), murine mediastinal stromal tissue (MST) and murine bone marrow (BM). The proliferating cells were characterized by fluorescence-activated cell sorting (FACS) using a panel of common marker molecules [13,14] and in the first instance by the performance of classical differentiation protocols [15,16].

The new MMS protocol was established on the basis of the "hanging drop" procedure used for embryonic stem cell differentiation and enables the analysis of aggregates containing the same cell number. Using the MMS as well as classical protocols we were able to demonstrate efficient multilineage differentiation of the isolated mesenchymal progenitor cells. An advantage though of the MMS protocol is the significant lower amount of cells needed for the differentiation test *in vitro*.

## Results

### Successful establishment of mesenchymal progenitor cell populations from different murine tissues

**Figure 1:**
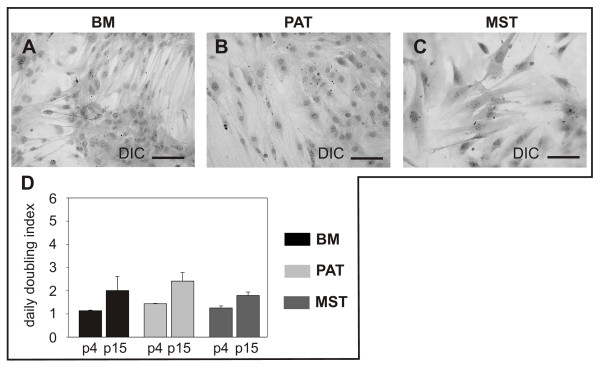
**Undifferentiated plastic-adherent cells derived from murine bone marrow (BM; A), perirenal adipose tissue (PAT; B), and mediastinal stromal tissue (MST; C) show a spindle-shaped morphology. **Comparison of proliferation of murine mesenchymal progenitor cells in passage 4 and 15 (D). Mean values ± SEM derived from independent samples per experimental group (n = 3) are shown. DIC = differential interference contrast. Bar = 100 μm.

Plastic adherent cell populations from BM, PAT and MST, obtained from NMRI mice, exhibited a typical spindle-shaped morphology (Fig. 1A-C). Isolation of murine progenitor cells from BM proved to be more difficult than from PAT and MST, since 80% of the BM isolates failed to persist in passaged cell cultures. However, two weeks after isolation in 20% of the BM isolates the predominant cell type changed and was replaced by a population of rapidly proliferating spindle shaped cells (Fig. 1A). In contrast, PAT and MST isolates displayed stable growth properties, with almost 100% successful rate of progenitor cell isolation.

Optimizing the cell culture for all murine progenitor cell isolates, ideal cell growth and maintenance was achieved using an initial cell concentration of 1 × 10^4 ^cells per cm^2^. All progenitor cell populations expanded *in vitro *and maintained stable growth properties in cell culture. Daily population doubling indices of all isolates were in the same range (Fig. 1D). Isolates cultivated for up to 15 passages did not show lack of proliferation after passaging or cryopreservation and did not show obvious chromosomal and karyotype abnormalities as indicated by sporadic samples for G- and C-banding (see Additional file 1). In higher passages (>16 passages) the cells stopped proliferating.

### Phenotypic heterogeneity of murine progenitor cells analyzed by flow cytometry

**Figure 2:**
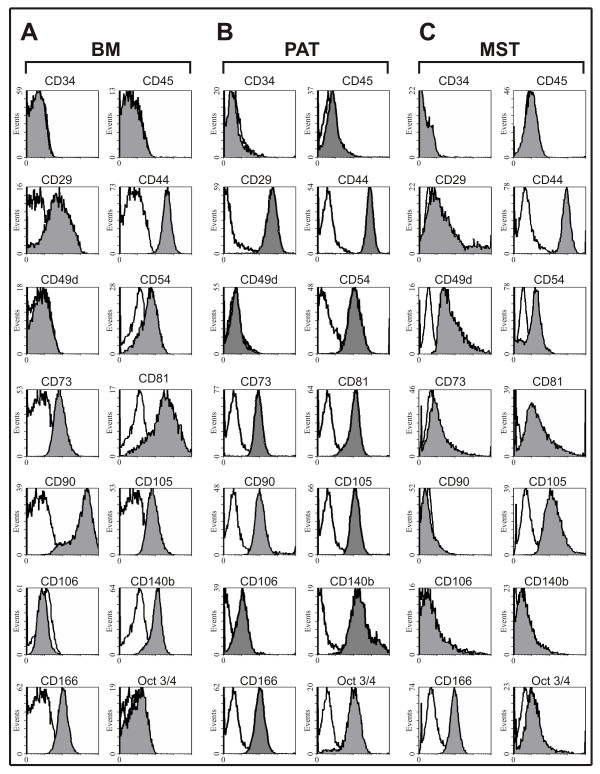
**Flow cytometric characterization of mesenchymal progenitor cells derived from murine bone marrow (BM; A), perirenal adipose tissue (PAT; B), and mediastinal stromal tissue (MST; C). **The background staining was assessed by isotype control (white). The specific markers are shown in grey.

To characterize the isolated mesenchymal progenitor cell populations, CD surface antigen marker expression was analyzed by means of flow cytometric measurement (Fig. 2). Murine BM, PAT, and MST cell isolates were negative for CD34 (gp105-120) and CD45 (leukocyte common antigen), indicating that they were not of hematopoietic origin (Fig. 2A-C). While all murine progenitor cell isolates displayed high degrees of CD44, CD81, CD105 and CD166, they showed differences in CD29 (MST-low), CD49d (MST-high), CD54 (PAT-high), CD73 (PAT-high; MST-negative), CD90 (MST-negative), CD106 (PAT-high), CD140b (MST-negative) and Oct 3/4 (PAT-high). Taken together, only MST isolates did not meet the minimal criteria of mesenchymal stromal cells [7] regarding marker molecule expression of CD73 and CD90.

### Chondrogenic differentiation of murine progenitor cells *via *micro mass body (MMB) and mesenchymal microsphere (MMS) cultivation

**Figure 3:**
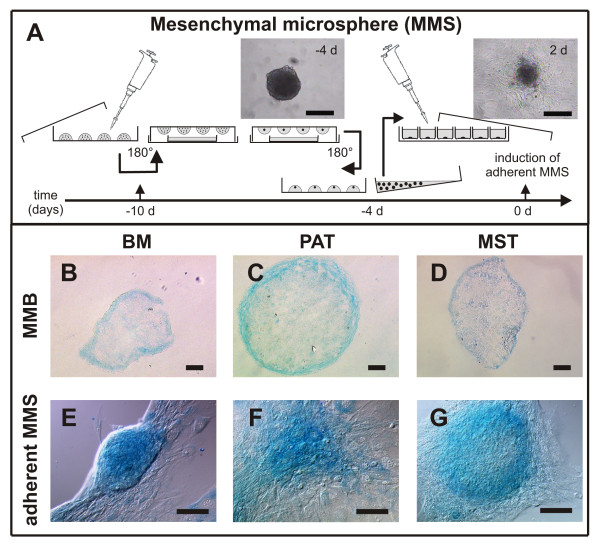
**After generation of mesenchymal microspheres (MMS) by hanging drop cultivation the standardized cellular aggregates are plated on plastic culture dishes (A); at 0 d specific medium is applied to induce differentiation and the MMS grow out. **Alcian blue (AB) staining (bottom) of cryosectioned "micromass bodies" (MMB) derived from murine bone marrow (BM; B), perirenal adipose tissue (PAT; C), and mediastinal stromal tissue (MST; D) as well as AB staining of MMS outgrowths (E-G) demonstrates chondrogenic differentiation of the mesenchymal progenitor cells with a maximum at day 25 of differentiation. Bar = 100 μm.

Differentiation of murine mesenchymal progenitor cells along the chondrogenic lineage using the classical MMB and new MMS protocol (Fig. 3A) was shown by AB staining (Fig. 3B-G). All MMBs and MMS cultured in chondrogenic medium stained positive for AB after 18 days of cultivation. Murine progenitor cell isolates from the three different sources displayed a comparable increase in the number of AB positive cells during further MMS and MMB cultivation (data not shown). After plating MMS grow out and vary in shape. While BM isolates often form nodular structures, PAT and MST outgrowths show a plane morphology.

**Figure 4:**
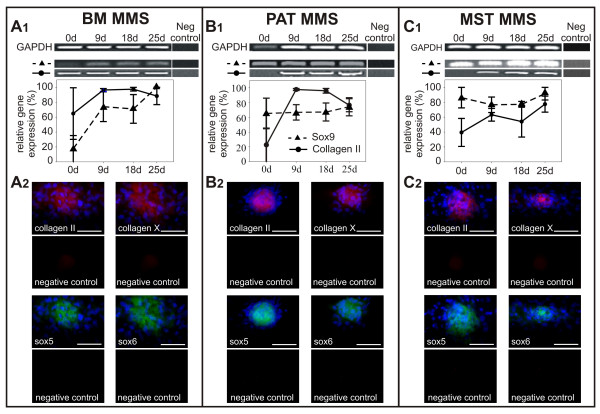
**Relative marker gene expression in MMS outgrowths derived from murine bone marrow (BM; A1), perirenal adipose tissue (PAT; B1), and mediastinal stromal tissue (MST; C1) confirmed chondrogenic differentiation of the mesenchymal progenitor cells. **Expression of collagen type II and X is demonstrated by immunostaining (A2, B2, C2; top) and *Sox5 *as well as *Sox6 *expression is shown by mRNA fluorescence *in situ *hybridization (A2, B2, C2; bottom). Nuclei are stained with DAPI (blue). Mean values ± SEM derived from independent samples per experimental group (n = 3) are shown. Bar = 100 μm.

RT-PCR analysis and immunostaining confirmed chondrogenic differentiation using the MMS method (Fig. 4). RT-PCR analysis for expression of chondrogenic marker genes demonstrated that all progenitor cell isolates expressed *collagen type II *as well as the associated transcription factor *Sox9 *during MMS differentiation (Fig. 4A1, B1, C1). In particular, in BM isolates *Sox9 *(Fig. 4A1*BM *(25 d): p ≤ 0,05) and *collagen type II *were up-regulated during chondrogenic differentiation, whereas *Sox9 *was continuously expressed in MMS derived from PAT (Fig. 4B1) and MST (Fig. 4C1) isolates. However, *collagen type II *was also up-regulated in PAT (Fig. 4B1*mPAT *(18 d): p ≤ 0,05) and MST isolates after induction of chondrogenesis. Immunostaining demonstrated that MMS from all isolates (Fig. 4A2, B2, C2) were positive for collagen type II and collagen type X at the end of a 25 day induction period. Taken together, these results demonstrate that all analyzed murine progenitor cell isolates were able to differentiate into the chondrogenic lineage using the MMS differentiation method.

### Osteogenic and adipogenic differentiation of murine progenitor cells *via *monolayer and mesenchymal microsphere (MMS) cultivation

**Figure 5:**
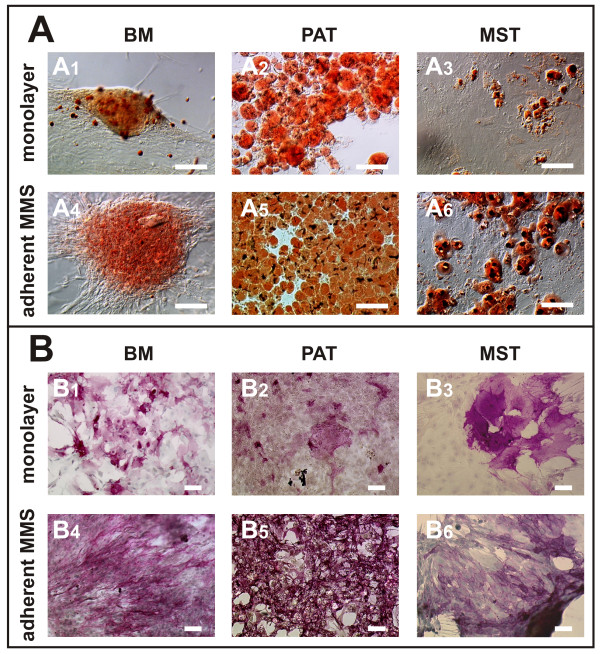
**Mesenchymal progenitor cells derived from murine bone marrow (BM), perirenal adipose tissue (PAT), and mediastinal stromal tissue (MST) show adipogenic (A; Sudan III staining) and osteogenic (B; alkaline phosphatase staining) differentiation. **Cells were cultivated *via *monolayer (A1-3 and B1-3) or adherent "mesenchymal microsphere" (MMS outgrowths; A4-6 and B4-6). Representative areas are shown. Bar = 100 μm.

**Figure 6:**
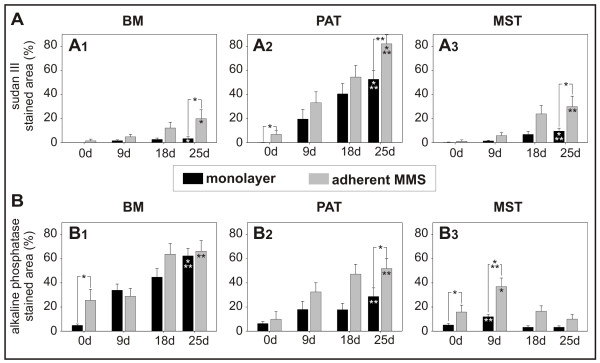
**Quantitative evaluation of adipogenic (A) and osteogenic (B) differentiation in murine bone marrow (BM; A1, B1), perirenal adipose tissue (PAT; A2, B2), and mediastinal stromal tissue (MST; A3, B3) cells cultivated *via *monolayer (black bars) and "mesenchymal microspheres" (MMS; grey bars). **Early differentiation by three-dimensional MMS prior to the application of specific induction media is represented at time point 0 d. Mean values ± SEM derived from independent samples per experimental group (n = 3) are shown. Significant differences are indicated: * = p ≤ 0,05; ** = p ≤ 0,01; *** = p ≤ 0,001.

To assess the adipogenic and osteogenic differentiation capacity of the analyzed murine adult mesenchymal progenitor cells, they were cultured in induction media as a monolayer [16,17] in comparison to three-dimensional MMS and characterized by histochemical staining (Fig. 5 and 6). Both, monolayer and MMS cultivation resulted in adipogenic (Fig. 5A) and osteogenic (Fig. 5B) differentiation of all analyzed murine progenitor cells. While MMS outgrowths derived from BM isolates showed a more nodular shape, the PAT and MST grow out plane.

Differentiation of monolayer cultivated BM, PAT, and MST progenitor cells with adipogenic induction medium resulted in a time-dependent increase of Sudan III positive droplets up to 25 d (Fig. 6; A1 *BM*: p ≤ 0,05; A2 *PAT*: p ≤ 0,05; A3 *MST*: p ≤ 0,001) and monolayer cultivation in osteo-inductive medium resulted in the appearance of AP positive cells (Fig. 6; B1 *BM *25 d: p ≤ 0,001; B2 *PAT *25 d: p ≤ 0,01; B3 *MST *9 d: p ≤ 0,01).

Similarly, in all MMS from BM, PAT and MST an increasing number of adipogenic lipid-laden cells up to 25 d (Fig. 6; A1 *BM*: p ≤ 0,05; A2 *PAT*: p ≤ 0,001; A3 *MST*: p ≤ 0,01) and an increasing number of AP positive cells could be detected during differentiation (Fig. 6; B1 *BM *25 d: p ≤ 0,01; B2 *PAT *25 d: p ≤ 0,01; B3 *MST *9 d: p ≤ 0,05).

When cultured *via *MMS, progenitor cells displayed changes in the differentiation efficiency which has not been observed in monolayer experiments. Cells from BM, PAT and MST differentiated *via *MMS into the adipogenic direction showed an almost two-fold increase in lipid-laden cells compared to monolayer cultivation. For example, approximately 80% of the MMS outgrowths derived from PAT stained positive for Sudan III after adipogenic induction for 25 days in contrast to 50% of the monolayer (Fig. 6A2*PAT *p ≤ 0,01). Likewise, the induction of BM and MST derived progenitor cells along the adipogenic lineage, resulted in a significant increase in lipid laden cells in comparison to monolayer culture (Fig. 6; A1 *BM *25 d: p ≤ 0,05; A3 *MST *25 d: p ≤ 0,05). Also osteogenic differentiation *via *MMS was enhanced in comparison to monolayer cultivation (Fig. 6; B2 *PAT *25 d: p ≤ 0,05; B3 *MST *9 d: p ≤ 0,001).

When cultivated *via *MMS, PAT derived progenitor cells differentiated into adipogenic cells prior to lineage specific induction, which could not be observed in monolayer experiments (Fig. 6A2*PAT *(0 d): p ≤ 0,05). Similar observations were made during MMS differentiation of cells from BM and MST into the osteogenic direction, demonstrating a higher number of AP positive cells prior to osteogenic induction (Fig. 6B1*BM *(0 d): p ≤ 0,05; 6B3 *MST *(0 d): p ≤ 0,05).

### Confirmation of osteogenic and adipogenic progenitor cell differentiation *via *MMS by RT-PCR and fluorescent immunostaining

To confirm the osteogenic and adipogenic differentiation of adult murine mesenchymal progenitor cells using the MMS protocol, we performed RT-PCR, analysing the expression of adipogenic and osteogenic marker genes.

**Figure 7:**
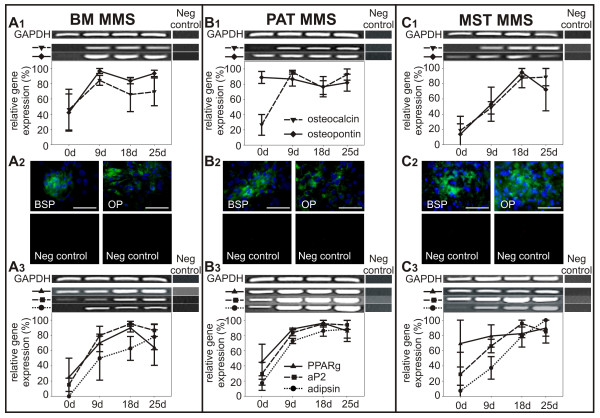
**Relative expression of marker genes in murine bone marrow (BM; A), perirenal adipose tissue (PAT; B), and mediastinal stromal tissue (MST; C) derived progenitor cells during osteogenic (A1, B1, C1) and adipogenic (A3, B3, C3) differentiation using the "mesenchymal microsphere" (MMS) protocol. **Expression of bone sialoprotein (BSP) and osteopontin (OP) is demonstrated by immunostaining in MMS (18 d; A2, B2, C2). Nuclei are stained with DAPI (blue). Mean values ± SEM derived from independent samples per experimental group (n = 3) are shown. Bar = 100 μm.

RT-PCR analysis of osteogenic marker genes showed that *osteopontin *was either expressed at a continuous level or up-regulated during MMS differentiation (Fig. 7A1, B1, C1). The expression of *osteocalcin *was initially up-regulated in BM (Fig. 7A1), PAT (Fig. 7B1*PAT *(9 d): p ≤ 0,01) and MST (Fig. 7C1*MST *(18 d): p ≤ 0,05) derived cells and continuously expressed during later stages of osteogenic differentiation. Immunostaining confirmed the expression of the osteogenic marker proteins bone sialoprotein and osteopontin in MMS (Fig. 7A2, B2, C2). Both proteins could be detected as early as 9 days post induction (data not shown) and were still expressed after 18 days of MMS cultivation.

Adipogenic induction of murine progenitor cells differentiated *via *the MMS protocol resulted in lineage-specific gene expression of marker molecules such as *adipsin*, *aP2 *and transcription factor *PPARγ *(Fig. 7A3, B3, C3). When cultivated *via *MMS, BM and PAT derived cells expressed transcription factor *PPARγ *at a basal level prior to lineage specific induction (Fig. 7A3, B3). Up-regulation of *PPARγ *in BM and PAT isolates was coherent with an increase in *aP2*-expression (Fig. 7; A3 *BM *(9 d): p ≤ 0,01; B3 *PAT *(9 d): p ≤ 0,05). Murine MST derived progenitor cells also displayed an up-regulation of *aP2 *and a stable expression level of *PPARγ *(Fig. 7C3). *Adipsin*, known to be a marker of mature adipocytes, was found to be expressed as early as day 9 in PAT (Fig. 7B3*PAT*: p ≤ 0,01) and continuously from the 18^th ^day of adipogenic differentiation *via *MMS in all analyzed progenitor populations (Fig. 7; A3 *BM*: p ≤ 0,01; C3 *MST*: p ≤ 0,01). Taken together, these results demonstrate that all analyzed murine progenitor cell isolates were able to differentiate into the adipogenic and osteogenic lineage using the MMS differentiation method.

### Comparison of cell numbers required for differentiation

To test the multilineage differentiation capacity of mesenchymal progenitor cells, we suggest a simple assay consisting of the characterization of adipogenic, chondrogenic and osteogenic differentiation at a single time point after application of specific induction media (e.g. in the time window from 9 d to 18 d, guaranteeing differentiation). According to our experience at least RT-PCR analysis and histochemical staining should be performed to get a reliable conclusion about the differentiation state of the cells.

Accepted that 1 × 10^4 ^cells/cm^2 ^are used for classical monolayer cultivation the following cell numbers are necessary to start adipogenic and osteogenic differentiation: (i) *442.000 cells *for RNA isolation (221.000 cells per adipogenic and osteogenic sample, respectively) on two 60 mm-plastic culture dishes (each dish with a surface area of 22,1 cm^2^) and (ii) *40.000 cells *for histochemical staining (per sample 20.000 cells) on 2 wells of one 2-well chamber slide (each well with a surface area of 2 cm^2^). Accepted that 200.000 cells/MMB are used to perform classical chondrogenic differentiation, *1.000.000 cells *are utilized for (i) RNA isolation (4 MMBs) plus (ii) histochemical staining (1 MMB). However, it has to be kept in mind that the generation of only one MMB for AB staining implies the high risk of loosing the sample due to technical problems during processing.

In fact, the introduction of the MMS method resulted in a significant decrease in the consumption of mesenchymal progenitor cells for testing the differentiation capacity in our experiments. To give an example, we again assume that the above mentioned simple screening test for differentiation is performed. Accepted that 5.000 cells/MMS are used the following cell numbers are now necessary to start differentiation: (i) for histocemical staining *75.000 cells *(15 MMS; 5 MMS per adipogenic, chondrogenic and osteogenic differentiation sample). (ii) *150.000 cells *are spent for RNA isolation (10 MMS per adipogenic, chondrogenic and osteogenic differentiation sample, respectively).

Taken together, in comparison to the new MMS method around 7 times more cells are wasted using the classical differentiation protocols. Again assuming that the above suggested screening assay is performed, in total *1.482.000 cells *(classical protocols) and, respectively *225.000 cells *(MMS protocol) are needed to start the differentiation. Moreover, the MMS cultivation resulted in comparable contents of RNA during adipogenic and osteogenic differentiation in comparison to monolayer cultivation (see Additional file 2). Although fewer cells are initially applied, chondrogenic differentiation *via *MMS resulted in a significant increase in the content of RNA in comparison to MMB cultivation. Finally, to give an idea about the time-saving effect of MMS cultivation, for example, in our experiments 1.482.000 cells were obtained in passage 4, whereas 225.000 cells could already be generated in passage 1.

## Discussion

It has been suggested that stem/progenitor cells capable of multilineage differentiation are distributed throughout the body [8]. We characterized plastic adherent murine cells from retroperitoneal PAT and thoracic MST as new tissue sources of mesenchymal progenitor cells and introduced the three-dimensional mesenchymal microsphere method for *in vitro *differentiation of stem/progenitor cells.

It is well accepted that adult stem cell isolation by classical protocols yields a heterogeneous population of stem/progenitor cells [9,11,10]. The frequency of colony forming unit-fibroblast among commonly used strains of mice has been observed to be highly variable [11,18]. Due to the low frequency of bone marrow derived stem cells their isolation has been an ongoing challenge [11]. In our study, 20% of the bone marrow isolates persisted in cell culture and demonstrated stable growth kinetics. In contrast, plastic adherent cells derived from PAT and MST demonstrated almost 100% successful isolates. Irregular anatomical distribution, donor variations and differences in sampling methods of stem/progenitor cells could influence the yield [19,20,7]. It has been suggested that initial plating densities ranging from 2-4 × 10^6 ^cells/cm^2 ^[11] to 1 × 10^2 ^cells/cm^2 ^[18] have a profound influence on stem/progenitor cell growth [21,22]. To standardize monolayer cultivation, we used a plating density of 1 × 10^4 ^cells/cm^2 ^for cells derived from PAT, MST, and BM. Providing these conditions, cell isolates showed stable growth kinetics as demonstrated by a stable population doubling index. No obvious chromosomal aberrations were observed in sporadic samples for G- and C-Banding. However, this karyotype analysis was not done systematically. Therefore, predominantly for higher passages of murine cells the possibility of chromosomal instabilities cannot be completely excluded.

It has been demonstrated that adult stem/progenitor cells express a panel of surface marker molecules [23,4,24]. In accordance with these observations we now show that all PAT, MST, and BM cell isolates were negative for CD34 and CD45, but expressed CD44, CD54, CD81, CD105, CD166. However, we observed that the expression of surface marker molecules differed among plastic adherent cells from different murine tissues. Murine MST-derived cells did not express CD29, CD73, CD90 and CD140b, while PAT and BM cell isolates expressed these markers. Furthermore, CD106 and Oct 3/4 were only expressed by PAT-derived progenitor cells. Thus, most of the analyzed marker molecules, characteristic for mesenchymal stem cells, were expressed by the PAT-derived cells. But both, BM and PAT isolates achieved the expression profile postulated by the current minimal criteria for mesenchymal stromal cells [7]. The expression of CD90, CD49d, and CD106, or lack thereof, has been a source of discrepancies in previous studies [4,23,18,13]. Taken together, the characterization by flow cytometry indicates that the analyzed cell isolates do not represent a homogeneous mesenchymal progenitor cell population. In addition, all isolates showed a fibroblastoid shape, but MST cells were larger. However, application of specific induction media following classical differentiation protocols resulted in an efficient differentiation of all isolates into the adipogenic, chondrogenic and osteogenic lineage.

The expression of Oct 3/4 is regulated during embryonic development, reflecting a role in specification and maintenance of pluripotent stem cells [25]. In accordance with our observations, it has been demonstrated that Oct 3/4 is expressed in adult stem/progenitor cell isolates [26] and in differentiated cell types [27]. In line with recently published results [28] we are reluctant to conclude that the expression of Oct 3/4 among a heterogeneous population of adult stem cells is indicative of pluripotency. Nevertheless, expression of this marker might indicate a more pronounced differentiation potency. This agrees with our observation that PAT-derived mesenchymal progenitor cells, which expressed Oct34, showed a higher differentiation capacity.

Johnstone et al. [15] introduced a three-dimensional model of MMB cultivation to assess chondrogenesis *in vitro*. Attempts to initiate chondrogenesis in monolayer cultures under otherwise similar conditions have failed [29,30]. The pellet culture format enables stem/progenitor cells to assume a close contact without spreading on a growth surface. In line with this, we predominantly found expression of cartilage matrix molecules in the centre of the MMS rather than in the outgrowing areas. We demonstrated chondrogenic differentiation *via *MMB as well as MMS for PAT, MST, and BM isolates. However, the classical MMB cultivation procedure takes a large amount of progenitor cells (200.000 cells/MMB) for differentiation and even classical adipogenic and osteogenic monolayer differentiation is often cell-consuming. Thus, considering the application of human stem/progenitor cells in medicine less cells can be provided for clinical application. Moreover, passaging for the generation of large amounts of cells results in a time delay prior to cell therapy.

## Conclusions

We introduced the MMS method to enable three-dimensional cell-cell-interactions during adipogenic and osteogenic progenitor cell differentiation using standardized conditions. MMS cultivation appears to improve the adipogenic differentiation capacity of PAT, MST and BM isolates and increased the yield of osteogenic cell differentiation in comparison to monolayer cultivation. Moreover, chondrogenic differentiation can also be analyzed using the MMS system. Taken together, we showed that the MMS system is feasible to test the *in vitro *differentiation capacity of plastic adherent cells. The main advantage of the MMS model system in this context is that only small amounts of mesenchymal progenitor cells are needed for testing differentiation. This cell-saving effect of the MMS model may result in less time consumption caused by the *in vitro *differentiation assay required prior to any clinical application of human stem/progenitor cells. Recently, the capability to form cellular aggregates has also been shown for human mesenchymal stem cells [31]. Therefore, MMS cultivation might be a useful tool to save human stem/progenitor cells for clinical application while testing their differentiation capacity.

## Methods

### Isolation of murine mesenchymal progenitor cells

All animals used for progenitor cell preparation were treated according to institutional guidelines. Preparation of murine progenitor cells was performed after cervical dislocation of NMRI mice (sex: female, age: 10 months; Charles River Laboratories Inc., Wilmington, DE, USA). Progenitor cells derived from PAT were obtained after laparotomy and surgical preparation of the retroperitoneal and perirenal tissue. Murine MST specimens were obtained after separation of the costo-sternal joints, luxation of the breast bone and surgical preparation of the anterior mediastinum. Murine PAT and MST samples were removed and separated from adjacent connective tissue. Tissue samples were digested with basal media supplemented with 1 mg/ml collagenase A (Sigma, München, Germany) for 45 min at 37°C, sieved, centrifuged, and washed with basal media. The isolation of BM derived stem/progenitor cells was performed by preparation of the lower extremities and exposition of the bone marrow cavity. Marrow cells were flushed out of the femur as well as the tibia using a 27-gauge needle with 10 ml of heparinised (5000 IE) basal media. The number of cells was determined and the cell suspension was plated onto 25 cm^2 ^tissue culture flasks (TPP, Trasadingen, Switzerland). The approximate number of cells obtained directly after isolation was 4 × 10^5 ^cells (PAT), 2.5 × 10^5 ^cells (MST) and 1.5 × 10^5 ^cells (BM). Non adherent cells were removed by the first medium change after two days. Single colonies of adherent fibroblast-like cells were first visible after 72 hours of cultivation. All cultivations were performed at 37°C and 5% CO_2_.

### Cultivation of murine mesenchymal progenitor cells

Progenitor cells were cultured in basal medium consisting of Dulbecco's modified Eagle's medium (DMEM, Invitrogen, Paisley, U.K.) supplemented with 1% sodium pyruvate (PAA, Pasching, Austria), 1% L-glutamine (PAA, Pasching, Austria), 1% MEM non-essential amino acids (Invitrogen, Paisley, U.K.), 1% penicillin/streptomycin (PAA, Pasching, Austria) and 10% fetal bovine serum (PAA, Pasching, Austria). Once adherent cells reached approximately 80-90% confluence, they were washed with phosphate buffered saline (PBS), trypsinized, and centrifuged for 5 min at 250 *g*. The cells were plated at a density of 1 × 10^4^/cm^2 ^and passaged every 4 to 10 days up to passage 15. Karyotype analysis in early (passage p5) as well as later (BM: passage p15 as well as PAT and MST: p13; see Additional file 2) stages was performed using standard methods for G- and C-Banding [32]. When reaching confluence, the cells were phenotyped using FACS analysis (passage p4) or replated for differentiation. Adipogenic, chondrogenic and osteogenic differentiation was analyzed in three independent samples (passages p5, p9 and p12) per experimental group in all three progenitor cell isolates. The daily doubling index was used to determine the proliferation and growth properties of the murine progenitor cells. Murine BM-, PAT- and MST-isolates were plated at a density of 1 × 10^4 ^cells per cm^2 ^and the increase in cell number was determined every 24 hours to calculate the daily doubling index.

### Differentiation of murine mesenchymal progenitor cells *via *classical "micro mass body" (MMB) and monolayer cultivation

Chondrogenic differentiation was performed using MMB cultivation. Cells were trypsinized, counted, and basal medium was replaced by chondrogenic induction medium. Aliquots of 2 × 10^5 ^cells in 0.5 ml chondrogenic induction medium were centrifuged at 65 *g *in 15 ml polypropylene conical tubes. Chondrogenic induction medium consisted of basal medium supplemented with 0.1 μM dexamethasone (Merk, Darmstadt, Germany), 300 μM ascorbic acid (Sigma, München, Germany), 1 mM L-proline (Sigma, München, Germany), 10 ng/ml transforming growth factor (TGF) β_3_, (R&D, Wiesbaden, Germany) and 1% ITS premix (Becton Dickinson, Heidelberg, Germany: 6.25 μg/ml insulin; 6.25 μg/ml transferrin; 6.25 μg/ml selenious acid; 1.25 mg/ml bovine serum albumin; 5.35 mg/ml linoleic acid). Samples of MMBs were taken for RNA-isolation (4 MMB per sample per day), hisotchemical or immunhistochemical analysis (1 MMB per sample per day) during the course of chondrogenic differentiation in three independent samples per experimental group (n = 3). MMBs prepared for histochemical and immunhistochemical staining were embedded in Tissue-Tek O.C.T. (Sakura Finetechnical, Tokyo, Japan), frozen at -80°C and cryosectioned (10 μm) for further analysis. To screen for proteoglycan deposits or marker protein expression within the chondrogenic MMBs, cryosections were fixed and stained with Alcian blue (AB) [33] or immunostained. Uninduced MMBs were stained as negative controls.

To analyse adipogenic and osteogenic differentiation, isolated progenitor cells were differentiated *via *monolayer protocols. Adipogenic and osteogenic induction of the progenitor cells was performed at 80-90% confluence. To induce osteogenic differentiation cells were treated with osteogenic medium for 25 days. Osteogenic medium consisted of basal medium supplemented with 0.1 μM dexamethasone (Merk, Darmstadt, Germany), 10 mM β-glycerolphosphate (Sigma, München, Germany) and 300 μM ascorbic acid (Sigma, München, Germany). To induce adipogenic differentiation cells were treated with adipogenic induction medium and adipogenic maintenance medium for 25 days. Induction medium consisted of basal medium supplemented with 0.5 mM 3-isobutyl-1-methylxanthine (IBMX Sigma, München, Germany), 1 μM dexamethasone (Merk, Darmstadt, Germany), 200 μM indomethacin (Sigma, München, Germany) and 2 μM insulin (Sigma, München, Germany). Following a four-day induction period, the adipogenic induction medium was replaced with adipogenic maintenance medium consisting of basal medium supplemented with 2 μM insulin for three days. This cycle was repeated three times and ultimately followed by a four-day period of adipogenic maintenance culture.

Lipid accumulation during adipogenic differentiation was demonstrated by Sudan III staining. Cells were washed with PBS followed by staining with a 0.2% solution of Sudan III (Sigma, München, Germany) in 70% ethanol. Alkaline Phosphatase (AP) activity of progenitor cells differentiating along the osteogenic lineage was demonstrated using protocols for fixation (2.5 ml citrate solution (Sigma, München, Germany), 6.5 ml acetone (Roth, Karlsruhe, Germany), 0.8 ml formaldehyde 37% (Merck, Darmstadt, Germany)) and AP staining (125 μl FRV-alkaline solution (Sigma, München, Germany), 125 μl sodium nitrite solution (Sigma, München, Germany) 125 μl naphthol AS-BI alkaline solution (Sigma, München, Germany), 5.63 ml *aqua dest*.).

### Differentiation of murine progenitor cells *via *three-dimensional "mesenchymal microsphere" (MMS) cultivation

To compare classical adipogenic and osteogenic monolayer and chondrogenic MMB differentiation with a cell-saving three-dimensional model of *in vitro *cell differentiation, isolated mesenchymal progenitor cells were differentiated *via *the MMS protocol (see Fig. 3A). For MMS cultivation 20 μl aliquots of a primary cell suspension at a density of 2.5 × 10^5 ^cells/ml were pipetted onto the bottom of a 100 mm bacteriological petri dish (GBO, Essen, Germany). By turning it upside down "hanging droplets" were obtained. To avoid evaporation, a lid of a 60 mm culture dish was filled with PBS and placed within the MMS cultivation chamber. The "hanging droplets" were cultured for six days until the formation of spheroids was visible. On the 6th day of MMS formation, cellular aggregates were flushed down with basal medium and collected using a 100 μl pipette. MMSs were plated onto 60 mm culture dishes (TPP, Trasadingen, Switzerland; 10 MMS per dish for total RNA isolation) and 2-well-chamber slides (B&D, Franklin Lakes, NJ, USA; 4 MMS per well for Sudan III, alkaline phosphatase and AB staining and for immunostaining, respectively). Four days after plating of the MMS, the spheroids firmly adhered and were differentiated using adipogenic, chondrogenic or osteogenic induction media as described above in three independent samples (passages p5, p9 and p12) per experimental group (n = 3). The time point of the application of induction media was denominated as 0 d. During the course of differentiation MMS remained adherent to the cell culture surface and a cellular outgrowth could be observed. The differentiation was analyzed at 0 d and at least at three different time points after induction medium was applied (9 d, 18 d and 25 d).

### Quantitative analysis of histochemical staining

To compare monolayer and MMS differentiation of mesenchymal progenitor cells derived from BM, PAT and MST by AP or Sudan III staining, ten areas of 0.77 mm^2 ^for osteogenic differentiation and ten areas of 0.235 mm^2 ^for adipogenic differentiation were quantified per sample per day in three independent samples per experimental group (n = 3). The stained areas were measured in relation to the total analyzed area of cells using ImageJ software (NIH, Bethesda, MD, USA) and quantified in percent.

### Fluorescent immunostaining

MMS cultured on chamber slides or MMB cryosections were rinsed three times with PBS, fixed for 5 min with pre-cooled (-20°C) methanol-acetone at 4°C, washed four times with PBS and incubated at room temperature for 30 min with 7.5% bovine serum albumin. Specimens were then incubated for 1 hour with a primary antibody in a humidified chamber at 37°C. Antibodies specific for the following proteins were used (designation, dilution ratio in PBS as well as reference are given in parentheses): collagen type II (II-II-6B3; 1:20; [34]), collagen type X (XAC9; 1:20; [35]), osteopontin (MPIIIB101; 1:20; [36]), bone sialoprotein I+II (WVID1(9C5); 1:20; [36]). The antibodies were obtained from the Developmental Studies Hybridoma Bank (University of Iowa, Iowa City, IA, USA). After rinsing four times with PBS, slides were incubated for 1 hour at 37°C with either fluorescein isothiocyanate (FITC, Dianova, Hamburg, Germany; 1:200) or cyanine3 (Cy3, Dianova, Hamburg, Germany; 1:600) labelled anti-mouse IgG as well as 4',6-Diamidino-2-phenylindole dihydrochloride (DAPI; Sigma, Taufkirchen, Germany). Slides were washed four times in PBS and briefly washed in distilled water. The method to couple immunostaining with fluorescence *in situ *hybridization has been previously described [37]. The probes used to detect *Sox5 *and *Sox6 *have been described elsewhere [38]. After immunostaining the specimens were embedded in Vectashield mounting medium (Vector, Burlingame, CA, USA) and analyzed with the fluorescence microscope Axioskop (ZEISS, Oberkochen, Germany). Negative controls were performed using only the secondary antibody. In addition, negative controls without the application of induction media were performed showing no differentiation (see Additional file 3).

### RT-PCR analysis

Mesenchymal progenitor cells differentiated *via *MMS or MMB were collected at different time points, washed twice with PBS, and total RNA was isolated using a standardized RNA Isolation Kit (Macherey&Nagel, Düren, Germany). The RNA concentrations were determined by measuring the absorbance at 260 and 280 nm. Samples of 500 ng RNA were reverse transcribed using oligo-dT primer and Superscript II reverse transcriptase following the manufacturer's recommendations (Invitrogen, Paisley, U.K.). Aliquots of 1 μl from the reverse transcriptase reactions were used for amplification of transcripts using primers specific for the analyzed genes and Taq polymerase according to the manufacturer's instructions (Fermentas, St.Leon, Germany). Reverse transcriptase reactions were denatured for 2 min at 95°C, followed by amplification for 30-40 cycles of 40 s denaturation at 95°C, 40 s annealing at the primer-specific temperature and 50 s elongation at 72°C. Primers specific for the following genes were used (sequence, annealing temperature, size as well as cycle numbers are given in parentheses): PPARγ (5'-GCC TAA GTT TGA GTT TGC TGT G-3', 5'-TGT CAT CTT CTG GAG CAC CTT-3', 58°C, 226 bp, 36), aP2 (5'-ATG CCT TTG TGG GAA CCT-3', 5'-GCT TGT CAC CAT CTC GTT TT-3', 58°C, 333 bp, 30), adipsin (5'-CTG ACA GCC TTG AGG ACG A-3', 5'-AGA GCC CCA CGT AAC CAC A-3', 58°C, 356 bp, 36), osteopontin (5'-TCA CTC CAA TCG TCC CTA CA-3' 5'-TGC TCA AGT CTG TGT GTT TCC-3', 58°C, 289 bp, 36), osteocalcin (5'-GCA GGA GGG CAA TAA GGT AG-3', 5'-CAG GGC AGA GAG AGA GGA CA-3', 58°C, 267 bp, 36), collagen type II (5'-ACG GTG GCT TCC ACT TCA-3', 5'-TAC ATC ATT GGA GCC CTG GA-3' 58°C, 383 bp, 35), Sox9 (5'-CTC TGG AGG CTG CTG AAC G-3', 5'-TTG TAA TCG GGG TGG TCT TTC TT-3', 60°C, 82 bp, 40), and GAPDH (5'-GGA AGG GCT CAT GAC CAC A-3', 5'-CCG TTC AGC TCT GGG ATG AC-3', 58°C, 164 bp, 30). Electrophoretic separation of PCR products was carried out on 2% agarose gels (2% (w/v) agarose (Roth, Karlsruhe, Germany), 0.7 ng/ml ethidium bromide (Roth, Karlsruhe, Germany)). The fragments were analyzed by computer-assisted densitometry in relation to GAPDH gene expression. The densitometric values of each marker were calculated in relation to GAPDH. From these values the highest of each marker was taken as 100%. Distilled water and no-RT reactions were always included as a negative control.

### Statistical Analysis

Statistical analysis was performed using SigmaPlot 2000 software (Systat, Erkrath, Germany) and calculated according to the student's t-test. Probes were analysed in three independent samples per experimental group (n = 3).

### Fluorescence activated cell sorting (FACS) of murine mesenchymal progenitor cells

Tyrpsin/EDTA- (0.25%) treated cells were washed twice with FACS buffer (PBS, 1% BSA and 0.1% NaN_3_) and adjusted to approximately 5 × 10^5 ^cells/ml and subsequently stained. A 100 μl cell suspension was incubated with 10 μl phycoerythrin (PE) conjugated monoclonal antibodies (mAbs), 10 μl of FITC conjugated mAbs, 10 μl of allophycocyanin (APC) mAbs or alternatively 10 μl non-conjugated mAbs and a secondary rat anti-mouse IgG-FITC at 4°C for 30 min. To discriminate mesenchymal progenitor cells from cells of hematopoietic origin, isolates were stained for CD34 and CD45. In addition, the following antigens were included to the phenotyping profile: CD29, CD44, CD49d, CD54, CD73, CD81, CD105, CD106, CD140b, CD166 as well as Oct 1/3. Prior to the FACS analysis, all samples were filled up to a total volume of 500 μl with FACS buffer. Cells were analyzed on a Cytomics FC 500 flow cytometer using cytomics CXP software (Beckman Coulter, Krefeld, Germany). At least 10,000 events were acquired and analyzed using a three parametric protocol (FL1, FL2 and FL4). Cell debris and aggregates were excluded by gating (FSC/SSC dotplot). Non-specific isotype-matched controls (IgG1, IgG2a, IgG2b, and IgM) were used to determine background fluorescence. All antibodies used were purchased from Becton Dickinson (Heidelberg, Germany), except CD105, CD166, and Oct 3/4 (RD Systems, Abingdon, U.K.) as well as CD34 and CD140b (eBioscience, San Diego, CA, USA).

## Author Disclosure Statement

All authors disclose any commercial associations and conflicts of interest, whether they are actual or potential. All authors state that no competing financial interests exist.

## List of Abbreviations

AB: alcian blue; AP: alkaline phosphatase; BM: bone marrow; MMB: micro mass body; MMS: mesenchymal microsphere; MST: mediastinal stromal tissue; PAT: perirenal adipose tissue

## Authors' contributions

F.B., A. G., J.R. and J.K. performed and analyzed the cell cultures and staining procedures. U.L. and P.S. established the flow cytometry for this study. M.M., J.R. and J.K. carried out RT-PCR analysis. H.L. has revised the manuscript critically and contributed to the analysis of the data. F.B., J.R. and J.K. designed the study and performed the statistical analysis. All authors read and approved the final manuscript.

## Supplementary Material

Additional file 1 The karyotype of mesenchymal progenitor cells from bone marrow up to passage 15 (A) as well as from perirenal adipose tissue (B) and mediastinal tissue (C) up to passage 13 is demonstrated by G-banding. Click here for file

Additional file 2 Monolayer and MMS cultivation result in comparable concentrations of RNA during adipogenic (A) and osteogenic (B) differentiation. In contrast to the MMB cultivation technique the chondrogenic differentiation of murine mesenchymal progenitors *via *the MMS system results in a continuous increase of the content of RNA (C). Mean values ± SED derived from at least three independent experiments (n = 3) are shown. Significant differences are indicated: * = p ≤ 0,05; *** = p ≤ 0,001. Click here for file

Additional file 3 **Description: **Mesenchymal progenitor cells derived from murine bone marrow (BM), perirenal adipose tissue (PAT), and mediastinal stromal tissue (MST) do not show differentiation *via *MMB (A) and MMS (B) without application of specific induction media. Expression of collagen type II and X as well as of bone sialoprotein (BSP) and osteopontin (OP) were analyzed by immunostaining, and these results serve as additional negative controls. Nuclei are stained with DAPI (blue). DIC = differential interference contrast. Click here for file
