# Supplementary figures and images for: Murine mesenchymal progenitor cells from different tissues differentiated via mesenchymal microspheres into the mesodermal direction
Source: BMC Cell Biol. 2009 Dec 19;10:92. doi: 10.1186/1471-2121-10-92 (PMC2809059; doi:10.1186/1471-2121-10-92)

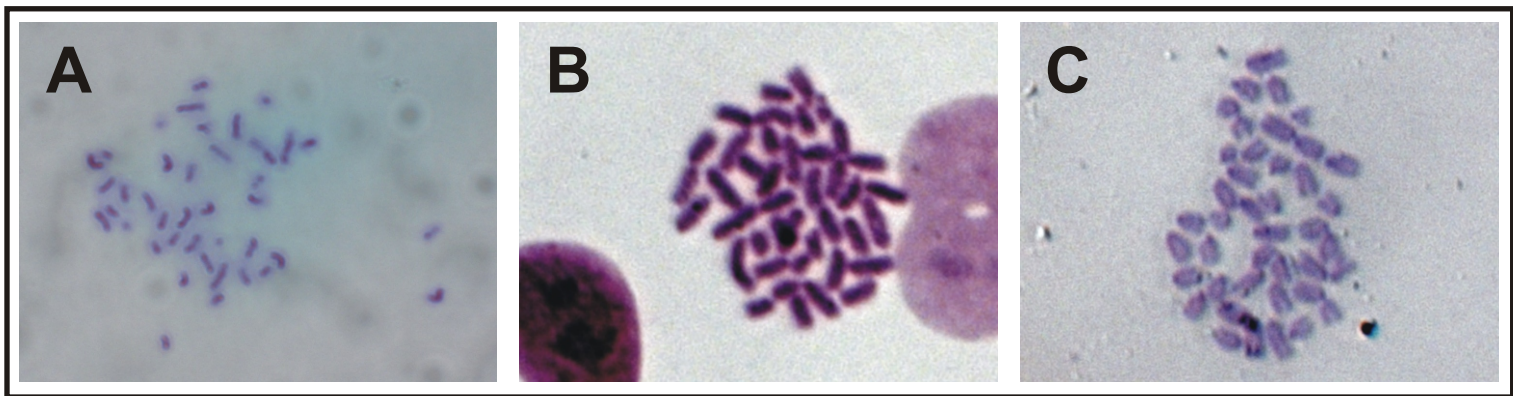

Supplement: Additional file 1 — The karyotype of mesenchymal progenitor cells from bone marrow up to passage 15 (A) as well as from perirenal adipose tissue (B) and mediastinal tissue (C) up to passage 13 is demonstrated by G-banding. [file 1471-2121-10-92-S1.PDF]

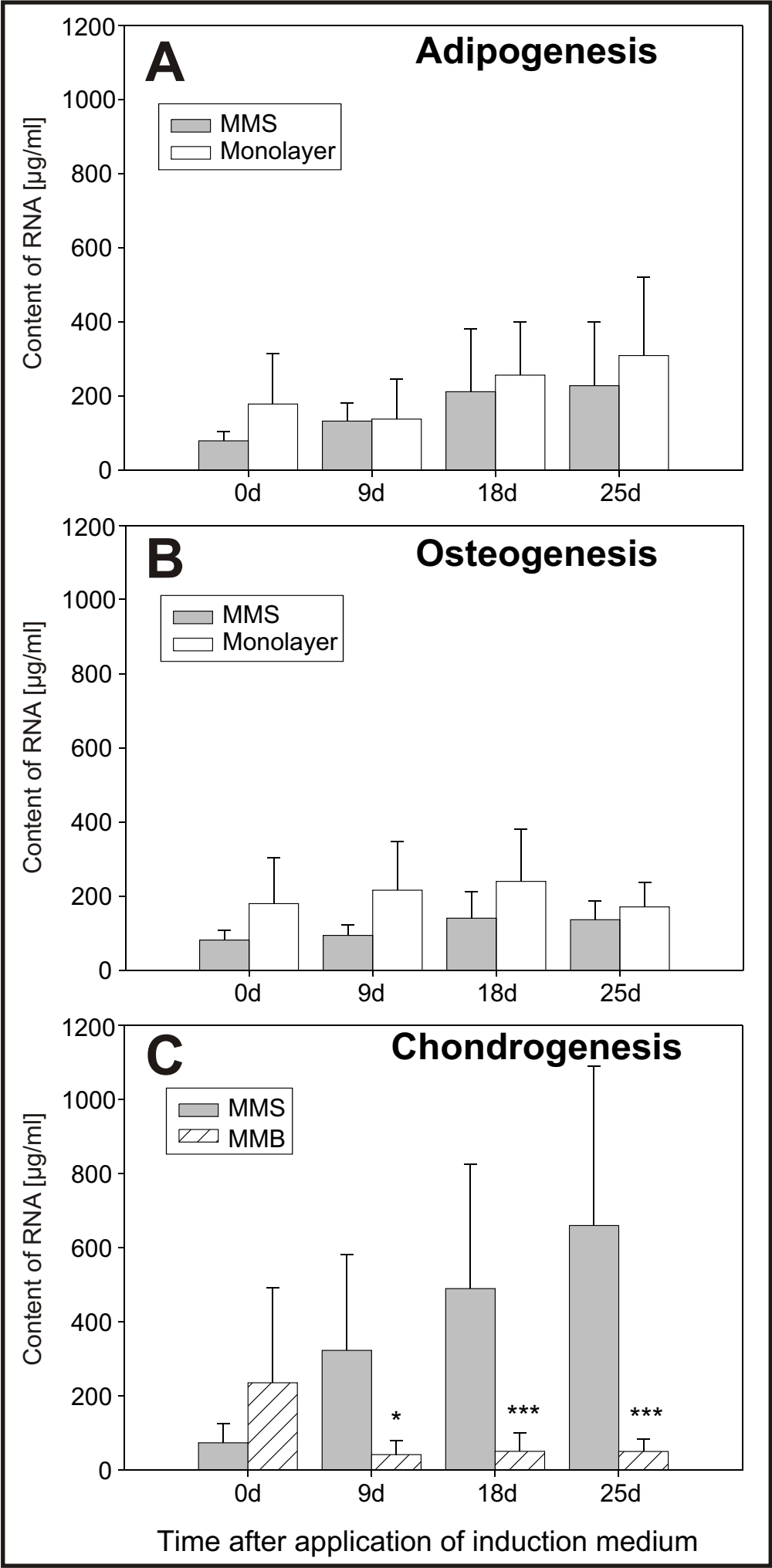

Supplement: Additional file 2 — Monolayer and MMS cultivation result in comparable concentrations of RNA during adipogenic (A) and osteogenic (B) differentiation. In contrast to the MMB cultivation technique the chondrogenic differentiation of murine mesenchymal progenitors via the MMS system results in a continuous increase of the content of RNA (C). Mean values ± SED derived from at least three independent experiments (n = 3) are shown. Significant differences are indicated: * = p ≤ 0,05; *** = p ≤ 0,001. [file 1471-2121-10-92-S2.pdf]
