# Supplementary material for: Murine mesenchymal progenitor cells from different tissues differentiated via mesenchymal microspheres into the mesodermal direction
Source: BMC Cell Biol. 2009 Dec 19;10:92. doi: 10.1186/1471-2121-10-92 (PMC2809059; doi:10.1186/1471-2121-10-92)
Supplement: Additional file 3 — Description: Mesenchymal progenitor cells derived from murine bone marrow (BM), perirenal adipose tissue (PAT), and mediastinal stromal tissue (MST) do not show differentiation via MMB (A) and MMS (B) without application of specific induction media. Expression of collagen type II and X as well as of bone sialoprotein (BSP) and osteopontin (OP) were analyzed by immunostaining, and these results serve as additional negative controls. Nuclei are stained with DAPI (blue). DIC = differential interference contrast. [file 1471-2121-10-92-S3.PDF]

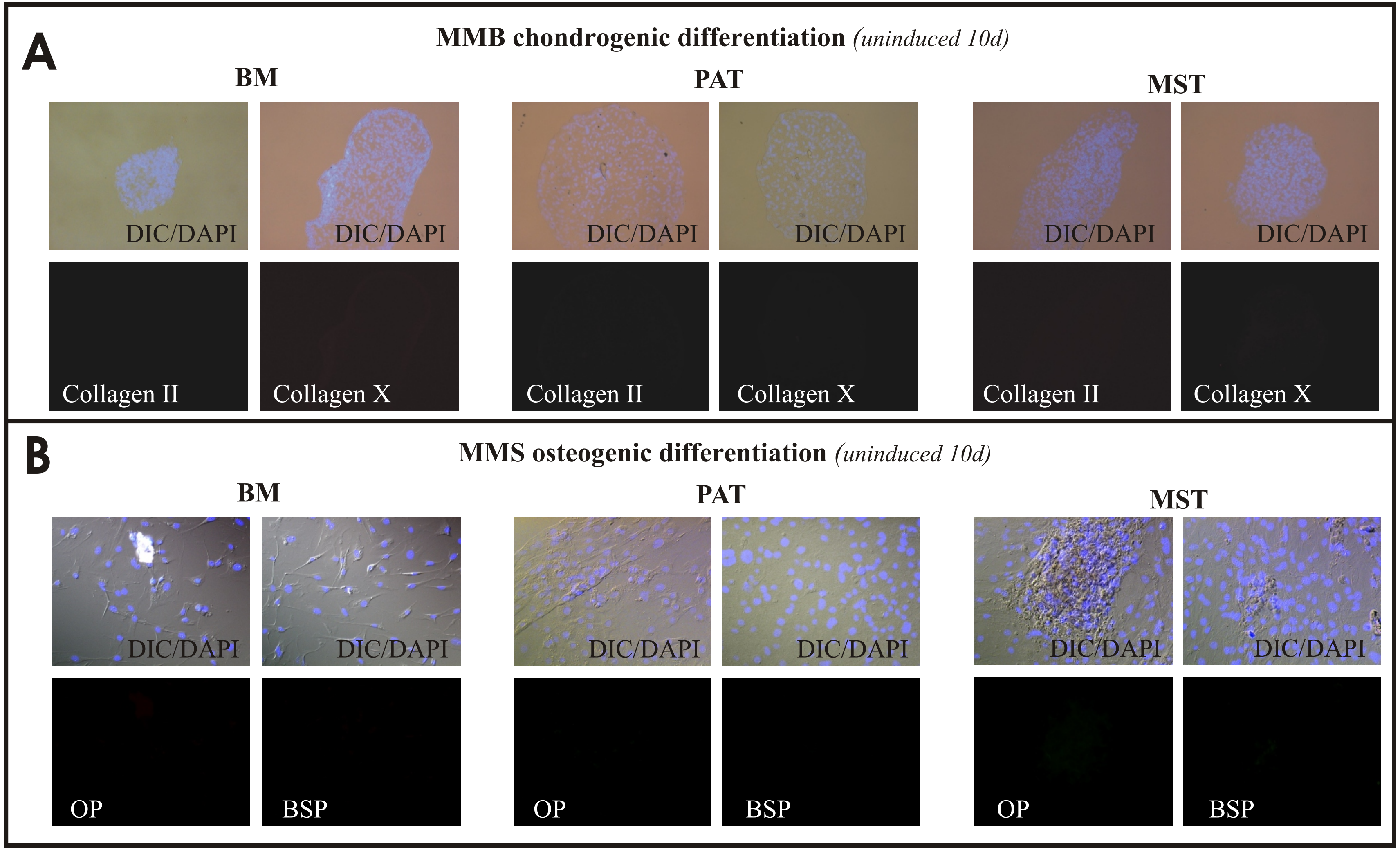
A
MMB chondrogenic differentiation (uninduced 10d)
BM
PAT
MST
DIC/DAPI
Collagen II
Collagen X
B
MMS osteogenic differentiation (uninduced 10d)
BM
PAT
MST
DIC/DAPI
OP
BSP
